# Akacid Medical Formulation Induces Apoptosis in Myeloid and Lymphatic Leukemic Cell Lines *In Vitro* and *In Vivo*

**DOI:** 10.1371/journal.pone.0117806

**Published:** 2015-02-13

**Authors:** Hannes Neuwirt, Elisabeth Wabnig, Clemens Feistritzer, Iris E. Eder, Christina Salvador, Martin Puhr, Zoran Culig, Petra Massoner, Martin Tiefenthaler, Michael Steurer, Guenther Konwalinka

**Affiliations:** 1 Department of Internal Medicine IV, Medical University of Innsbruck, Innsbruck, Austria; 2 Department of Internal Medicine V, Medical University of Innsbruck, Innsbruck, Austria; 3 Department of Experimental Urology, Medical University of Innsbruck, Innsbruck, Austria; 4 Department of Pediatrics, Medical University of Innsbruck, Innsbruck, Austria; 5 Department of Internal Medicine I, Medical University of Innsbruck, Innsbruck, Austria; National Cheng Kung University, TAIWAN

## Abstract

Akacid medical formulation (AMF) is an oligoguanidine that exerts biocidal activity against airborne and surface microorganisms including bacteria, viruses, fungi, and molds, while showing relatively low toxicity to humans. We have previously shown that AMF exerts antiproliferative effects on a variety of solid tumor cell lines. In this study we raised the question whether AMF could also substantially inhibit cell growth or induce apoptosis in cell lines derived from hematologic malignancies such as leukemia or lymphoma. We found that AMF has antiproliferative effects on various hematologic cell lines derived from human leukemia and lymphoma. Additionally, we show that AMF induces apoptosis in leukemia cell lines not only via the extrinsic and intrinsic pathway, but also in a caspase-independent manner. This effect was found also in G0-arrested cells. Finally, in our animal experiments utilizing male nu/nu Balb/c mice we found a significant growth retardation, which was immunohistochemically associated with a significantly lower number of KI67-positive cells and caspase-3 induction in AMF-treated mice.

## Introduction

Akacid medical formulation (AMF) is an oligoguanidine that exerts biocidal activity against airborne and surface microorganisms including bacteria, viruses, fungi, and molds [1], while showing relatively low toxicity to humans [2]. We have previously shown that AMF exerts antiproliferative effects on a variety of solid tumor cell lines [3]. Additionally, we found that this was associated with reduced phosphorylation of mitogen-activated protein kinases (MAPK, ERK) 1 and 2 in prostate cancer cell lines. Furthermore, this was accompanied by downregulation of cell cycle regulators such as cyclin D1, cyclin-dependent kinase -2 and -4 in prostate cancer cells [3]. Since then, no study has been published further elucidating possible anti-tumor effects of AMF.

As we have had already conducted a study on solid tumor cell lines, we raised the question whether AMF could also substantially inhibit cell growth or induce apoptosis in cell lines derived from hematologic malignancies such as leukemia or lymphoma.

Recent advances in targeted therapy against leukemia and lymphoma using, for instance, monoclonal antibodies have led to improved remission rates and better patient survival. Chemotherapeutic drugs currently in use such as alkylating agents, direct inhibitors of DNA synthesis or synthetic nucleoside analogues exert a direct cytotoxic effect by various mechanisms. However, the precise mechanism of action of many of these substances still remains to be elucidated. It is still not clear how hydroxycarbamide, for instance, used in the treatment of hematological neoplasms, exerts its effects on neoplastic cells.

In any case, induction of apoptosis is one of the major goals of chemotherapy in all kinds of malignant tumors. Apoptosis is induced either by the extrinsic or the intrinsic pathway, for which caspase 8 and 9, respectively are known to be the up-stream effectors. Both pathways merge into the downstream effector capase-3, which finally induces apoptosis including fragmentation of DNA or poly (ADP-ribose) polymerase (PARP) cleavage, the products of which (cPARP) are generally used for assessing apoptosis induction. The extrinsic pathway is usually activated via binding of the so-called death-receptors to their ligands, for instance, the death receptor CD95 (Fas) which binds to its ligand namely the FasL. The intrinsic pathway, on the other hand, is considered as non-ligand-dependent but is induced by direct damage of cell proteins or DNA by ionizing radiation or cytotoxic chemicals. The first step in the intrinsic apoptosis pathway is the release of mitochrondial cytochrome c into the cytoplasm, which subsequently activates caspase-9. In order to modulate both apoptosis induction pathways, we used bcl-2, a commonly known and strong inhibitor of the intrinsic pathway (cytochrome c release) and CrmA, a poxvirus protein, to inhibit FasL-induced apoptosis (extrinsic pathway) [4,5].

In the present study we found that AMF has antiproliferative effects on hematologic cell lines. Additionally, we show that AMF induces apoptosis in leukemia cell lines not only via the extrinsic and intrinsic pathway, but also in a caspase-independent manner. This effect was seen even in G0-arrested cells. Finally, in our animal experiments we found a significant growth retardation, which was immunohistochemically associated with a significantly lower number of KI67-positive cells and caspase-3 induction in AMF-treated mice.

## Materials and Methods

### Chemicals

AMF was kindly provided by Geopharma (Vienna, Austria). It is prepared by polycondensation of equimolar amounts of guanidine hydrochloride and 1,2-bis(2-aminoethoxy)ethane. The original 19% stock solution, stored light-protected at room temperature, was diluted with phosphate-buffered saline (Dulbecco´s PBS (1x) without Ca & Mg, No. H15–002, PAA Laboratories GmbH, Vienna) to a 1% solution with a concentration of 10^–4^M and stored protected from light at 4°C.

Activating anti-Fas-antibody was purchased from Upstate (Upstate Cell Signaling Solutions, NY). Dexamethasone and N-Acetylsphingosine (C2-ceramide) were purchased from Sigma-Aldrich (Vienna, Austria). The cell permeable pan-caspase inhibitor benzyloxycarbonyl-Val-Ala-Asp-fluoromethylketone (ZVAD-FMK) was purchased from Calbiochem (Darmstadt, Germany).

### Cell lines

HL-60 (acute promyelocytic leukemia), K-562 (chronic myelogenous leukemia), U-937 (histiocytic lymphoma), TIB-152 (acute T cell leukemia), CRL-2319, CRL-2362 and CRL-2323 (peripheral blood normal B-lymphoblasts) were purchased from ATCC (Rockville, MD); CEM C7H2 (acute T cell leukemia) and its subclones (CEM 2E8 and 6E2) were kindly provided by Prof. Reinhard Kofler (Tyrolean Cancer Research, Innsbruck, Austria) and have been published previously [6,7,8,9,10]. All cell lines were maintained in RPMI-1640 (PAA-Laboratories, Vienna) and supplemented with 10% fetal bovine serum, glutamine and antibiotics (100 U/ml penicillin, 10 μg/ml streptomycin, 0.25 μg/ml amphothericin-B), all from Gibco (Life Technologies, Carlsbad, CA). Cell viability was assessed regularly before plating in experiments with CEM 6E2 using trypan blue staining.

### 3^H^-thymidine incorporation

The detailed protocol has been published previously [3]. In brief, cells were seeded in 96-well plates and incubated with AMF for the indicated time periods. Twelve hours before the intended time period, 2 μCi/well of 3H-thymidine was added; 12 hours later, cells were frozen at -18°C, thawed as needed and DNA was harvested on fiberglass filters. Quantification was performed using a liquid scintillation counter (Wallac 1410, Pharmacia, Uppsala, Sweden).

### Flow cytometry

We used fluorescein isothiocyanate (FITC)—labeled annexin V and counterstaining with propidium iodide (Annexin V-FITC Apoptosis Detection Kit, Alexis Biochemicals) according to the manufacturer´s protocol. Cell analysis was carried out with FACS-Calibur (Becton Dickinson, San Jose, CA). In brief, cells were harvested after AMF-treatment for the indicated time periods and 10^5^ cells were transferred into flow cytometry tubes. After washing with 1 ml PBS and centrifugation, 200 μl of annexin V-FITC was added to each tube and incubated for 10 minutes in the dark. Just before analysis, propidium iodide was added to each tube.

For cell cycle analysis cells were collected, washed with PBS, and stained with propidium jodide using CycleTest Plus, DNA Reagent Kit (Becton Dickinson, San Jose, CA) as previously published [3]. Cell cycle status was analysed on a Becton Dickinson Flow Cytometer (FACS Calibur). Gating strategies using FL3-W channel were applied in order to exclude doublets from the analysis.

### Caspase activation assays

We performed Caspase-Glo 3/7, Caspase-Glo 8 and Caspase-Glo 9 assay (Promega Corporation, Madison, Wisconsin) after treatment of cells with AMF for the indicated time periods according to the manufacturer´s protocol. In brief, CEM and HL 60 cells were seeded in black-walled 96-well plates. Following incubation, the plates were allowed to equilibrate to room temperature for 30 min, before addition of 50 μl of Caspase-Glo Reagent (Promega) to each well. Blank controls consisting of reagent and cell culture medium were always included as well as no-treatment controls consisting of reagent and cells in culture. The blank control was used to measure background luminescence associated with the culture medium and Caspase-Glo Reagent, whereas the no-treatment control was important for determining the basal protease activity of the cell culture system. In order to reduce the amount of non-specific background signal, we used the proteasome inhibitor MG-132 (Z-Leu-Leu-Leu-CHO) which was included in the assay kit. Thirty minutes after adding the reagent, luminescence was recorded using a plate-reading luminometer (Chameleon Microplate Reader, Hidex, Turku, Finnland). Background luminescence (blank control) was subtracted from each set of cell data, and fold-increase in activity was calculated based on activity measurement of untreated cells (no-treatment control). All samples were assayed in triplicate.

### Western blots

A detailed protocol has been published previously [3,11]. In brief, cells were incubated with different concentrations of AMF for the indicated time periods. Cells were then collected, washed with PBS and lysed. In all experiments, whole cell extracts were used. Fifty μg of protein per lane were then resolved using a gradient Bis-Tris gel (Invitrogen, Leek, The Netherlands) and transferred onto a nitrocellulose membrane (Invitrogen). After blocking for 1 hour using Starting Block (TBS) buffer (Pierce Biotechnology, Rockford, IL), primary antibody was added and incubated overnight at 4°C. Fluorescence-labeled secondary antibodies (Molecular Probes, Eugene, OR) were used. The membranes were scanned and quantified using the Odyssey infrared imaging system (LiCor Biosciences, Lincoln, NE). Antibodies were purchased from the following companies: beta-actin (Chemicon Int., Temecula, CA), cleaved PARP (Promega, Madison, WI), pro- and cleaved caspase-3 (Upstate Cell Signaling Sources, NY), pro- and cleaved caspase-8 (Naga-ku Nagoya, Japan), pro-caspase-9 (Cell Signaling Technology Inc, Beverly, MA).

### Animal experimentation

Male nu/nu Balb/c mice were kept in strict accordance with the institutional and governmental guidelines in the animal quarters of the University of Innsbruck and the current study has been approved by the ministry of science and research (Bundesministerium für Wissenschaft und Forschung—BMWF, permit number: BMWF-66.011/0001-II/10b/2008). All efforts were made to minimize suffering.

Aseptic surgery was performed after analgosedation with ketamine/xylazine for the implantation of jugular vein catheters. The catheter assembly consists of a guide cannula (Plastics One, Roanoke, VA) which is connected to a piece of silicone (Silastic) tubing that can be surgically inserted and fixed to the jugular vein. All surgical tools and supplies were autoclaved prior to surgery. After surgery, animals were allowed a minimum of 4–7 days of recovery. They showed no signs of distress after implantation. Then 1x10^6^ cells were 1:1 mixed with Matrigel (Becton Dickinson, San Jose, CA) of which 100 μl were injected subcutaneously into the right flank of the mice. Tumors were allowed to develop for approximately 1 week. AMF or vehicle treatment was started as soon as tumors were visible. AMF was applied via jugular vein catheters at a concentration of 5 mg/kg of body weight (adjusted to 25 μl) together with a 50 μl bolus of normal saline solution (0.9% NaCl) (total volume 50 μl per day). Vehicle- treated mice were administered 50 μl normal saline solution per day. In total, vein catheters were implanted in 20 mice of which five (25%) died the day after surgery. Fifteen mice were injected with tumor/matrigel mixture. Tumor take was achieved in 10 mice (66%), which were randomized into two groups (AMF treatment and vehicle treatment). During treatment, two mice (one from each group) had to be withdrawn from the experiment due to catheter occlusion. In total, eight mice completed a 2-week treatment, four with AMF and four with vehicle. Tumor size was measured regularly using a caliper and tumor volume was calculated using the following formula (tumor volume = length x width^2^). At the end of the experiment mice were killed after analgosedation with ketamine/xylazine by decapitation and tumors were harvested for further analysis.

### Immunohistochemistry

Immunohistochemical staining of xenograft tumors was done on formalin-fixed and paraffin embedded 4-μm sections using the Ventana autostainer model Discover XT (Ventana Medical System, Tucson, AZ) with an enzyme-labeled biotin streptavidin system and solvent-resistant 3,3`-diaminobenzidine Map kit. Slides were pretreated with tris borate EDTA buffer (pH 7.8, Roche) for 48 minutes. The following antibodies were used: Ki-67 (mouse monoclonal antibody, DAKO, Denmark), and cleaved caspase-3 (rabbit polyclonal antibody, Cell Signaling). Specificity of staining was controlled by including an unspecific control antibody (DAKO). Slides were counterstained with hematoxylin (Roche). Expression was evaluated manually. Number of Ki-67/cleaved caspase-expressing cells was determined by counting the number of positive cells. The numbers stated in the graphs are means of 10 visual fields at a magnification of 200-fold.

### Statistics

For each treatment group, statistical distribution was determined using Kolmogorov-Smirnov test. Because of non-Gaussian distribution, nonparametric tests were applied as follows. To assess the overall significance for experiments with more than one treatment group, we used the Kruskal-Wallis test. To confirm statistically significant findings in the Kruskal-Wallis test, Mann-Whitney U test was applied. For statistics on tumor growth in animal experiments, 2-way-ANOVA was used. P values < 0.05 were defined as statistically significant and marked with an asterisk (*). All statistical analyses were performed using SPSS 12.0 software (SPSS, Chicago, IL).

## Results and Discussion

### AMF inhibits cellular growth in myeloid and lymphatic cell lines

**Fig 1:**
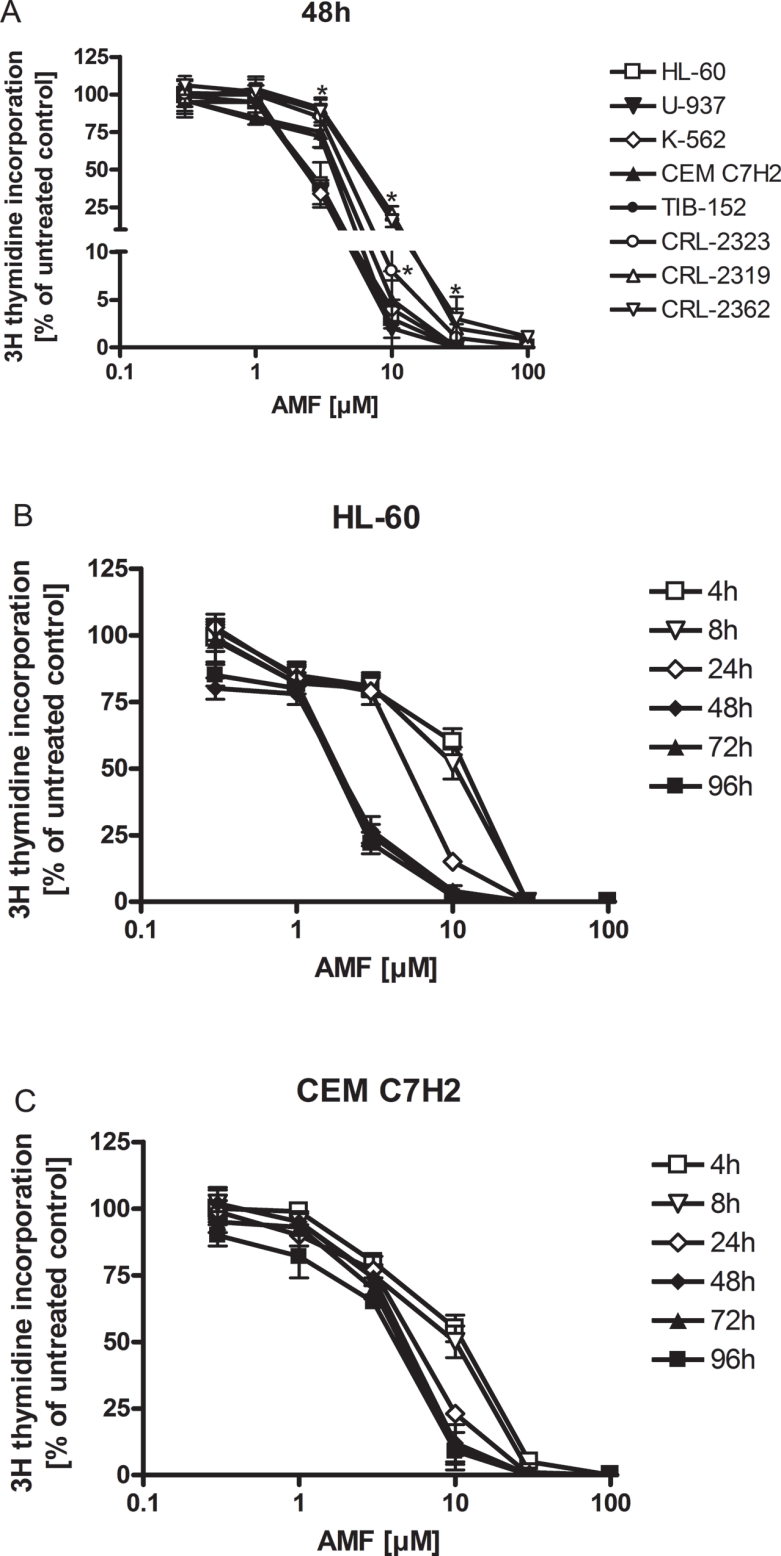
3H-thymidine incorporation is reduced in hematologic cell lines. Cells were treated with AMF for the indicated time periods with different concentrations. Proliferation is inhibited in a concentration- and time-dependent manner. Cell lines derived from normal lymphoblasts showed significant less sensitivity to AMF. n = 6 (except CCRL-2323, CRL-2319, CRL-2362 and TIB-153: n = 3) (number of independent experiments carried out in triplicates).

In order to assess a possible antiproliferative effect on hematologic cell lines, we treated HL-60 (acute promyelocytic leukemia), K-562 (chronic myelogenous leukemia), U-937 (histiocytic lymphoma) and CEM C7H2 (T- acute lymphatic leukemia) with various concentrations of AMF (0.3–100 μM) for 48 hours and measured 3H-thymidine incorporation. As shown in Fig. 1A, AMF significantly reduced cellular growth at concentrations of 3 μM in HL-60, K-562 and U-937 (IC50 2.3 μM). CEM C7H2 and TIB-152 cells, which were less sensitive, were significantly inhibited at 10 μM with an IC50 of 3.8μM and 3.5μM, respectively. Three normal peripheral blood lymphoblasts (CRL-2319, CRL-2362 and CCRL-2323) derived cell line were less sensitive with an IC50 of 5.5μM (CRL-2323) and 6.5μM (CRL-2319, CRL-2362). Although, the difference between malignant and CRL-2323 did not reach statistical significance except comparing proliferation at 10μM, the other two cell lines used showed significant less sensitivity to AMF at 3–30μM (marked with an asterisk). E.g. the 3H-thymidine incorporation rate was 5-fold higher at 10μM of AMF (25% vs. 5%) in the latter two normal cell lines compared with malignant ones. We found no difference at concentrations below 3μM, as these did not alter proliferation significantly in any cell line, as well as at 100μM, which was used in order to include a concentration of maximum toxicity (and was not used for further experiments).

In order to assess the time course of AMF effects, HL-60 and CEM C7H2 cells were treated with AMF for 4 to 96 hours. Short time periods (24, 8 and 4 hours) caused a time-dependent decrease of AMF effects on 3H-thymidine incorporation. In both cell lines, incubation longer than 48 hours did not yield additional growth retardation. These results are comparable to those derived from solid tumor cell lines, which have been previously published by our group [3].

### AMF induces time- and concentration-dependent apoptosis in HL-60 and CEM C7H2 cells

**Fig 2:**
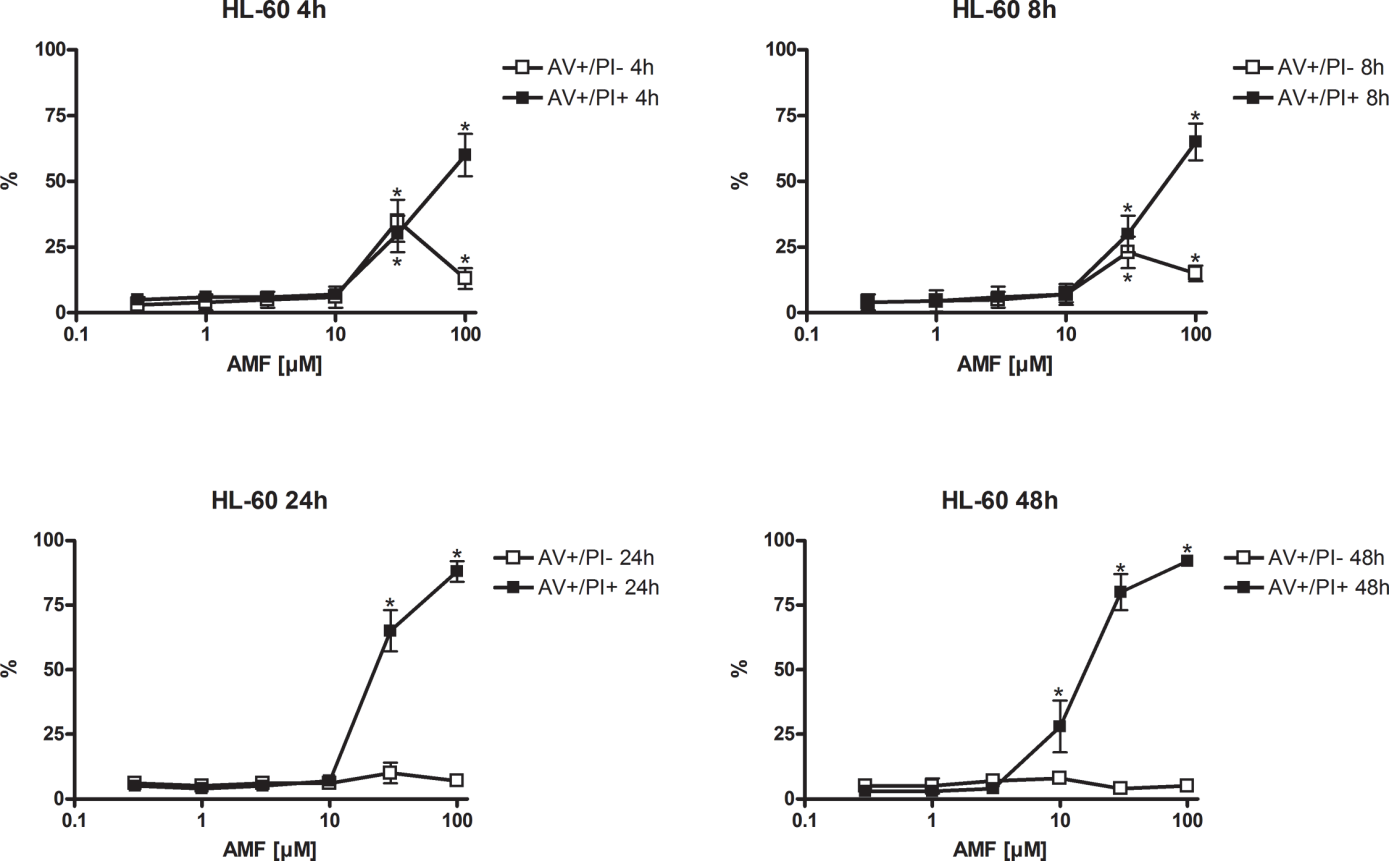
AMF induced apoptosis in HL-60 cells. After incubation with AMF for the indicated time periods Annexin-V and propidiumiodide staining was performed and analysed by flow cytometry. n = 4 (number of independent experiments carried out in triplicates).

**Fig 3:**
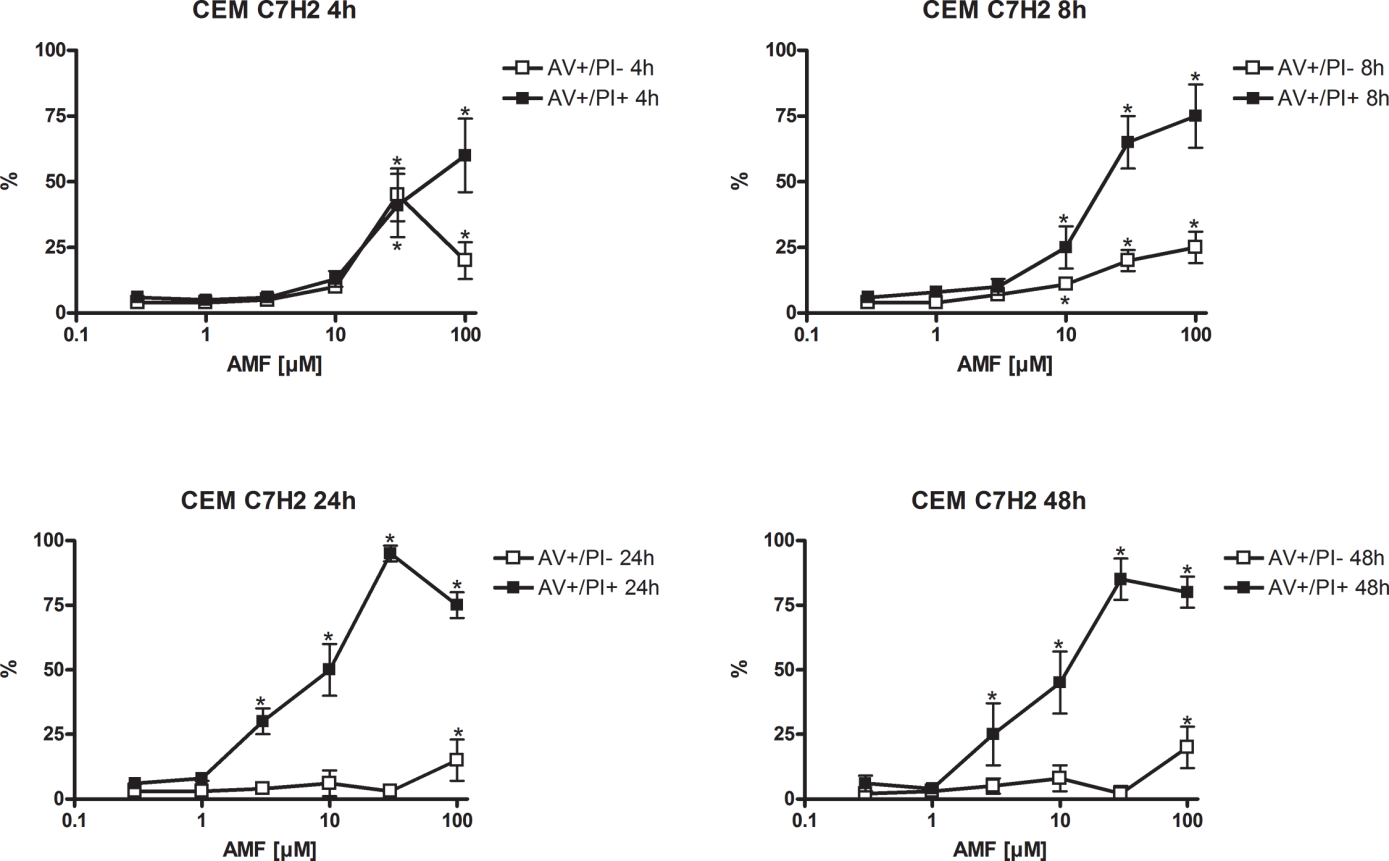
AMF induced apoptosis in CEM C7H2 cells. After incubation with AMF for the indicated time periods Annexin-V and propidiumiodide staining was performed and analysed by flow cytometry. n = 4 (number of independent experiments carried out in triplicates).

Next we raised the question if induction of apoptosis could be one reason for the decrease in proliferation. In order to assess early (AV+/PI-) and late apoptosis (AV+/PI+), we labeled cells using an anti-Annexin-V (AV) (FITC-labeled) antibody and/or propidium iodide (PI). HL-60 and CEM C7H2 cells were treated for 4 to 48 hours with different concentrations of AMF. As shown in Fig. 2, AMF caused a time- and concentration-dependent induction of apoptosis with a peak in early apoptotic cells already after 4 hours treatment with 30 μM AMF. The percentage of AV+/PI- cells decreased with prolongation of treatment, whereas AV+/PI+ cells steadily increased with time and concentration of applied AMF to almost 100%. Similar results were found in CEM C7H2 cells (Fig. 3). Interestingly, although less sensitive to AMF in proliferation compared to HL-60 cells, apoptosis was already induced at 3 μM in CEM C7H2 cells. Up to now, no one has investigated the effects of AMF on HL-60 and CEM cells. That CEM cells are more sensitive to apoptosis induction compared to HL-60 has been demonstrated using herbal extracts [12] and synthetic molecules [13].

### Induction of apoptosis is associated with increased activity of the intrinsic and extrinsic apoptotic pathway

**Fig 4:**
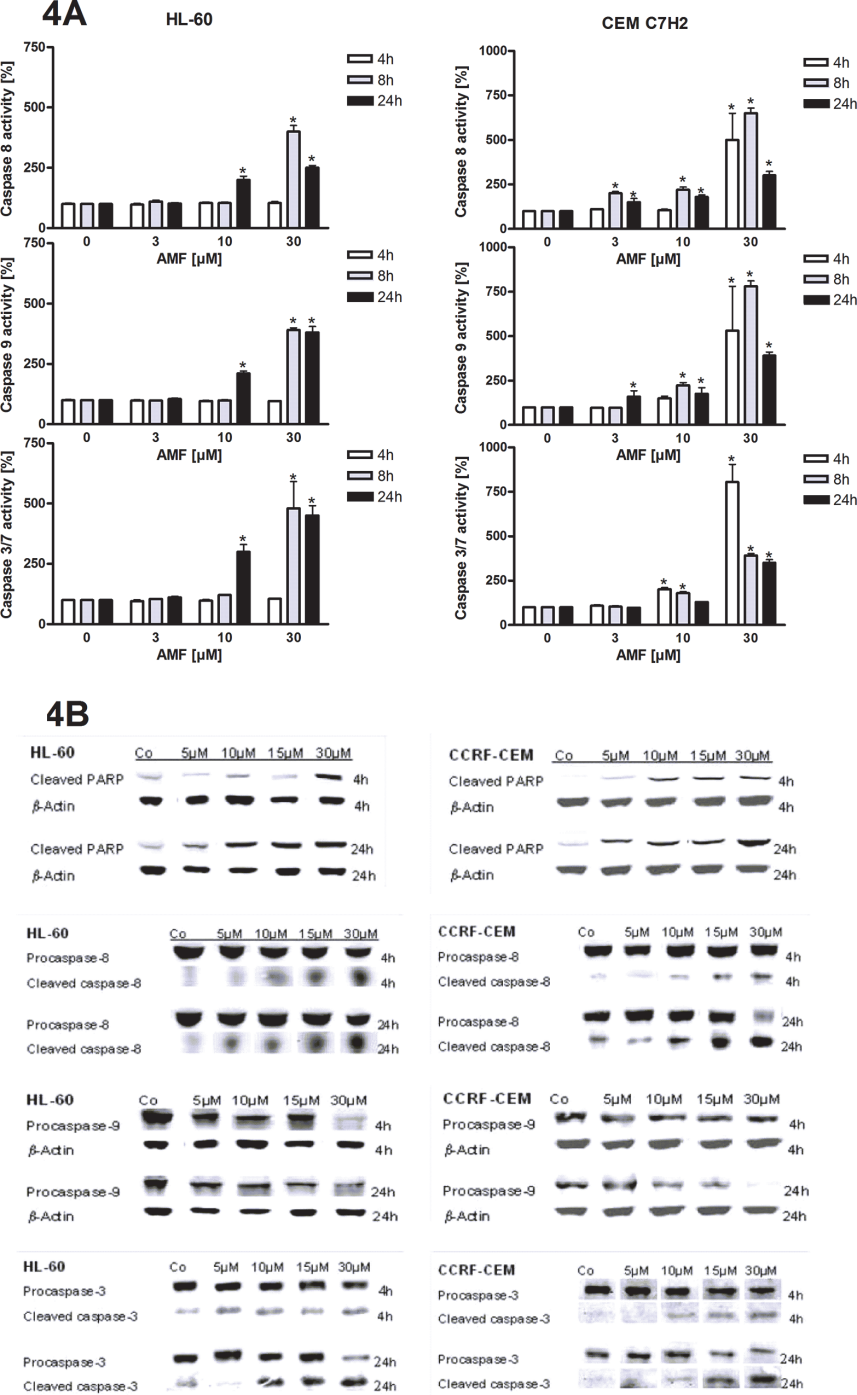
Caspases are activated after AMF treatment. Capase -8, -9, and -3/-7 activity was measured using a luminometric assay after treatment with various concentrations of AMF for different time periods (4A). n = 3 (number of independent experiments carried out in triplicates). Furthermore Western blot analyses were performed for caspase activation and additionally for cleaved PARP, as a marker for apoptosis induction (4B). n = 3 (number of independent experiments, respresentative blots are shown)

In order to investigate whether the extrinsic or intrinsic apoptosis pathway was involved in AMF-induced apoptosis, we assessed caspase 8, -9 and -3/-7 activity using luminometric assays. As shown in Fig. 4A, AMF induced activity of all tested caspases in a concentration- and time-dependent manner. In particular, caspase 8 was induced already 8 hours after application of 3 μm of AMF in CEM C7H2, whereas in HL-60 higher concentrations and a longer treatment (10 μM for 24 hours) were necessary to yield a similar effect. Analogous results were found for caspase 9 activity. Finally, also caspase 3/-7 activity was induced in both cell lines, with CEM C7H2 being the more sensitive one. These results were corroborated on protein basis utilizing Western blotting for cleaved PARP, caspase -3, -8 and procaspase-9 (Fig. 4B). Unfortunately, we had to stick to an anti-procaspase-9 antibody (due to technical reasons), which when cleaved/activated should be decreased at protein level. However, as anticipated we found a profound decrease of procaspase-9 levels, suggestive for activation, which was found as stated above in experiments for Fig. 4A. Hence, we conclude that AMF induces apoptosis via both the extrinsic and the intrinsic pathway, which has been found to be the case also with various naturally occurring and synthetic molecules [14,15,16].

### Induction of apoptosis does not depend on caspase activation

**Fig 5:**
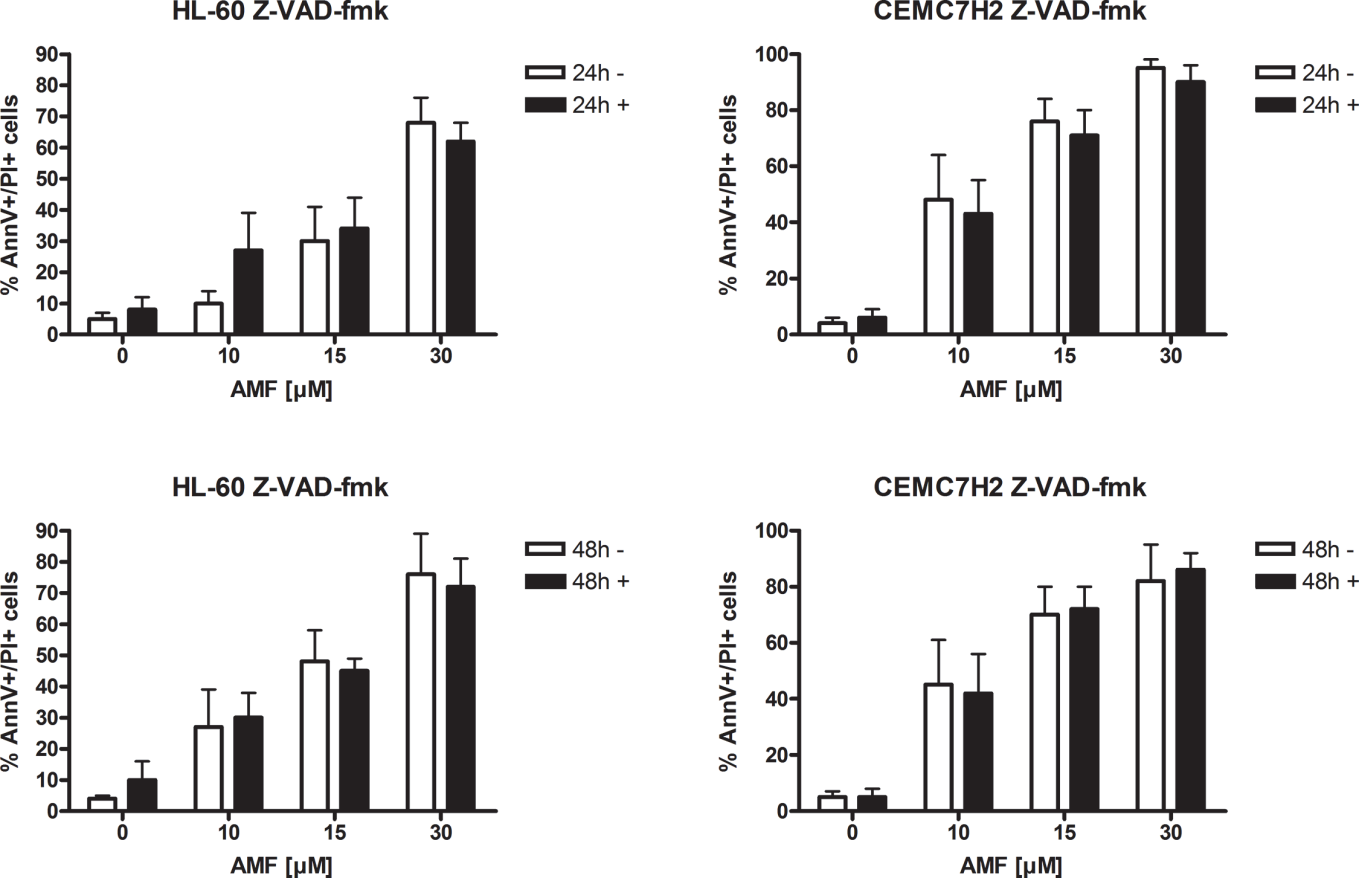
Apoptosis is also induced in presence of a pan-caspase inhibitor. Cells were preincubated with Z-VAD-fmk, a pan-caspase inhibitor, before AMF treatment and apoptosis induction was assessed using Annexin-V and propidiumiodide staining and flow cytometry. n = 4 (number of independent experiments carried out in triplicates).

Next we raised the question if AMF-induced apoptosis was only associated with activation of caspases or causally linked to it. To answer this question, we pretreated HL-60 and CEM C7H2 cells with 20 μM of the pan-caspase inhibitor Z-VAD-fmk before addition of AMF and AV/PI labeling. In order the test the cellular system, a FAS-ligand (FASL) control was also included, as it has been shown that Z-VAD-fmk significantly inhibits FASL-induced apoptosis (see S1 Fig.). After 24 and 48 hours of AMF treatment (10–30 μM), preincubation with pan-caspase inhibitor did not significantly reduce induction of apoptosis in neither of the two cell lines tested (Fig. 5). These results strongly suggest that AMF-induced apoptosis does not depend only on caspase activation. Similar results have been shown in various cell lines using, for example, magnolol (a small-molecule hydroxylated biphenol) [17], alpha hydroxy acid [18] and distillation remnants from Japanese liquor [19]. Additionally, it has been shown that chemotherapeutics like topoisomerase II inhibitors, kinase inhibitors and proteasome inhibitors may induce apoptosis also by a caspase independent pathway possibly depending on Panx1 expression, which is a structural component in gap junctions and hemichannels an involved in nucleotide release during chemotherapeutic-drug induced apoptosis [20]. Matsumoto et al. also reported of a small-molecule inhibitor of glioma-associated oncogene 1 and -2 treatment induced caspase-independent apoptosis in a Ewing´s sarcoma cell line, which was associated with decreased expression of survivin, cyclin A and increase of p21 [21]. Another study published by Wang et al. showed that a citrus 5-acetyl-tetramethoxyflavone induces apoptosis in breast cancer cells via both caspase-dependent and –independent pathways, the latter being mediated by translocation of apoptosis inducing factor (AIF) into the nucleus [22,23]. Similar results have been reported in glioma cells after tetrandrine (a bis-benzylisoquinoline alkaloid) treatment [24]. Cordycepol C, a sesquiterpene isolated from a fungus species named cordyceps ophioglossoides, induced caspase-independent apoptosis in human hepatocellular carcinoma cells [25]. Similarly, Ashour et al. found that Rottlerin, a polyphenole isolated from Kamala trees, induced caspase-independent apoptosis [26]. The proposed mechanism of apoptosis induction by Cordycepol C and Rottlerin again was nuclear translocation of AIF.

**Fig 6:**
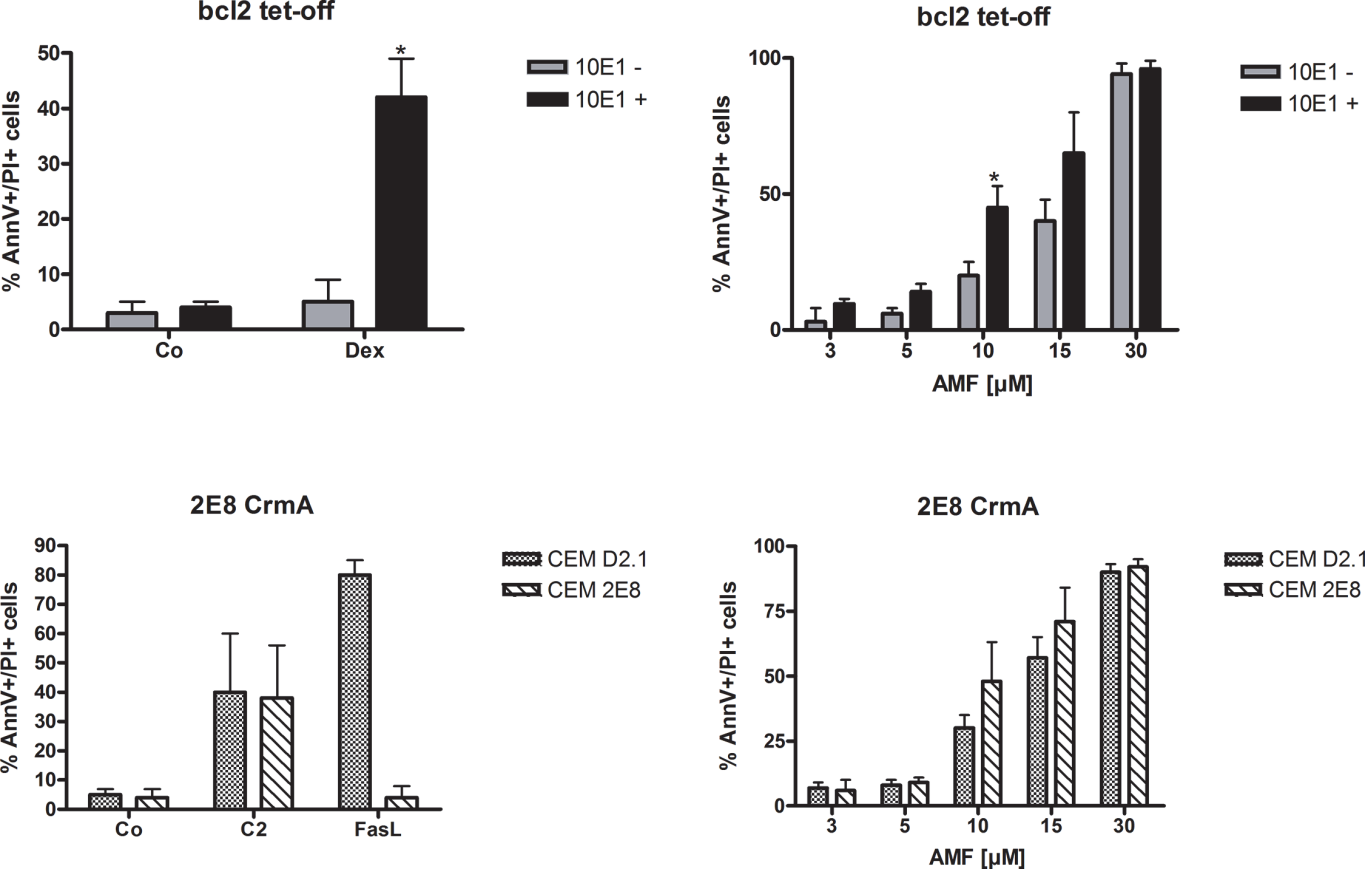
Bcl-2 and CrmA overexpression does not significantly inhibit AMF induced apoptosis. Subclones of CEM C7H2 cells expressing either CrmA (CEM 2E8) or tetracycline-regulated bcl-2 (tet-off, CEM 10E1, - without + with tetracycline) and the respective control subclone (CEM D2.1) were treated with AMF and Annexin-V and propidiumiodide staining was analyzed after 48hours of incubation. n = 4 (number of independent experiments carried out in triplicates).

In order to further test this hypothesis, we used stably transfected CEM C7H2 clones (10E1) that express bcl-2 (intrinsic apoptosis pathway involving caspase 9), which can be downregulated by addition of doxycycline via a tetracycline-responsive element (tet-OFF) as previously published [9]. In order to functionally test this cellular system, CEM 10E1 were treated with doxycycline (bcl-2 knock-down) or without it (bcl-2 expression) and treated with 0.1 μM of dexamethasone, a potent inducer of apoptosis in these cells. As shown in Fig. 6, (upper left panel) downregulation of bcl-2 expression by doxycycline (10E1+) significantly enhanced sensitivity to dexamethasone-induced apoptosis (see S2 Fig.). However, bcl-2 expression did not prevent AMF-induced apoptosis in this cellular model, except at 10 μM AMF (Fig. 6, upper right panel and S4 Fig.). Especially bcl-2 has become a focus of recent cancer research. For example, ABT-737, a bcl-2/bcl-X_L_ inhibitor, induced cell death even at nanomolar concentrations in lung cancer stem cells [27]. In acute lymphoblastic leukemia, especially the bcl-2 dependent early T-cell progenitor subgroup are very sensitive to ABT-199 induced apoptosis in vitro and in vivo, a selective bcl-2 inhibitor [28]. Similar findings have been published in HL-60 cells, lymphoma and acute myeloid leukemia cell lines [29,30,31]. Furthermore, another member of the BCL-2 family, mcl-1 has been shown to be essential for cellular growth of c-Myc-driven mouse lymphomas [32]. Preliminary clinical data also demonstrate that targeting apoptosis resistance with ABT-199 might overcome chemotherapy-refractory disease in bcl-2 overexpressing neoplasms such as follicular lymphoma [33] and in CLL patients with deleted/mutated TP53 [34]. Hence, further development of pro-apoptotic drugs such as AMF represents an attractive therapeutic strategy.

**Fig 7:**
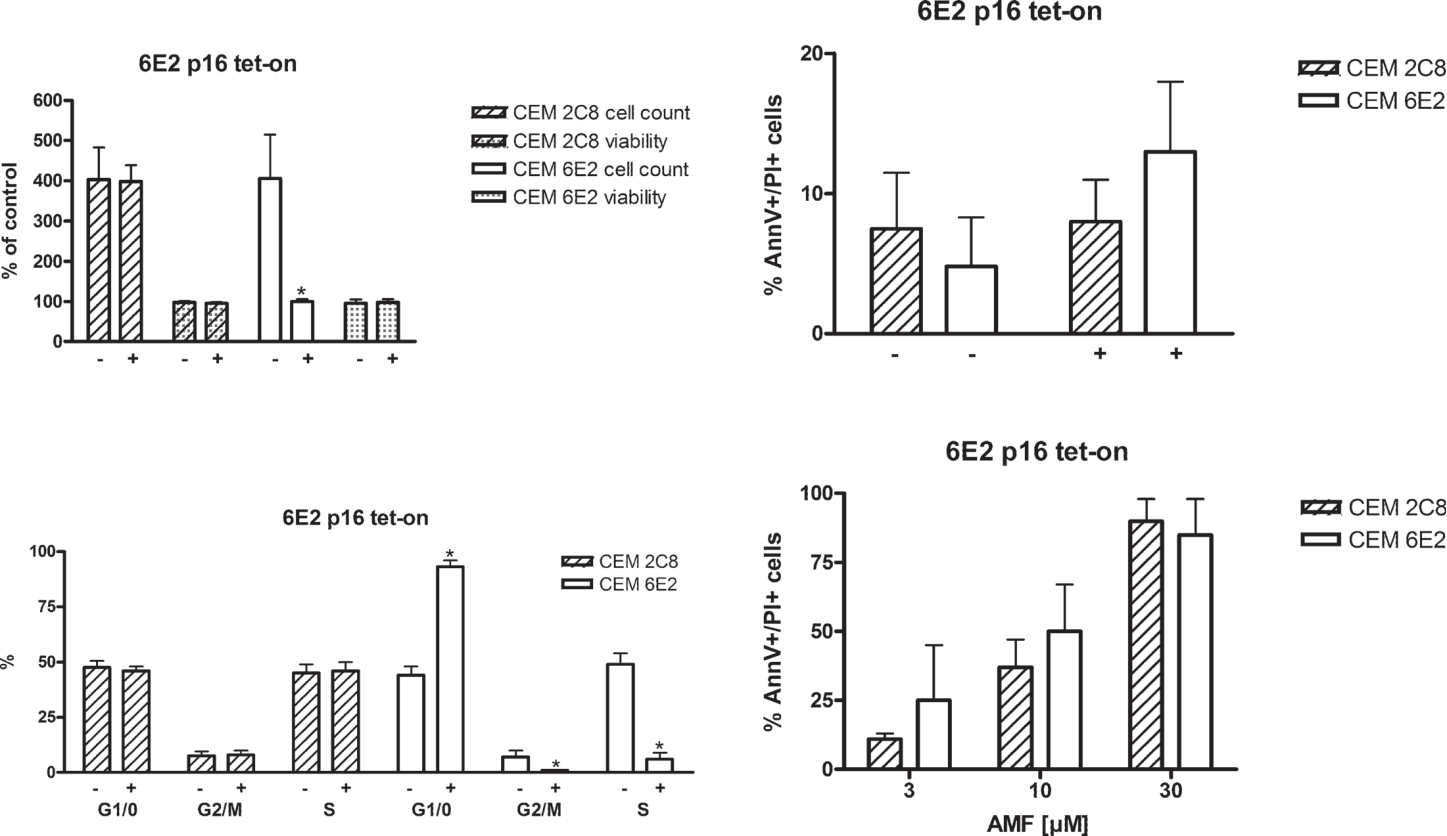
Apoptosis is also induced in G1/0 phase arrested cells. The CEM 6E2 subclone, which expresses tetracycline-regulated p16INK4A (CEM 6E2) and an empty vector transfected control cell line (CEM 2C8) were treated with tetracycline (+) or not (-). Cell viability (trypan blue staining), cell count (manual counting), as well as cell cycle distribution and apoptosis (Annexin-V, propidium iodide) were assessed. % of control for cell viability and cell count refers to the viability / cell number at seeding, which was set to 100%. n = 4 (number of independent experiments carried out in triplicates).

Next, we utilized a CEM C7H2 subclone that stably overexpressed CrmA (2E8). CrmA is a cowpox virus serpin that inhibits Apo1/Fas-induced apoptosis (extrinsic pathway involving caspase 8) [7,8]. The CrmA-negative subclone D2.1 served as control. The functionality of the cellular system was tested, as previously published [7,8], using C2-cermides, which induce apoptosis albeit CrmA expression, and FasL, for which it is known that CrmA potently inhibits apoptosis induction (Fig. 6, lower left panel and S3 Fig.). Similar to bcl-2, overexpression of CrmA did not diminish the percentage of apoptotic cells after treatment with AMF (Fig. 7, lower right panel and S4 Fig.). The expression of FAS and sensitivity to chemotherapy of leukemia cells has been investigated in vivo only in a very limited number of studies. One of which showed that in AML-cells FAS expression correlated significantly to response to initial induction chemotherapy [35].

Taken together, AMF has a strong pro-apopotic effect on the tested cell lines and although we did not further investigate the molecular mechanism one might hypothesize that—similar to synthetic and natural poly- and oligomers discussed above—AMF influences bcl-2 action and / or AIF translocation to the nucleus. Further studies are needed in order to finally clarify the exact mechamism of apoptosis induction by AMF.

### AMF induces apoptosis also in G1/0 arrested cells

In order to assess the potency of AMF also in non-dividing cells, which are known to be more resistant to antiproliferative substances, we used another CEM C7H2 subclone (6E2), which expresses the cell cycle inhibitor p16^INK4A^ under a tetracycline-responsive promoter (tet-ON) [6]. The pβrTA-plasmid transfected subclone 2C8 served as control [10]. As shown in Fig. 7, cell proliferation was substantially inhibited by the addition of doxycycline to CEM 6E2 cells, without alteration of cell viability (Fig. 7, upper left panel) or induction of apoptosis (Fig. 7, upper right panel). Flow cytometry revealed the functionality of this cellular system as we found an increase in G1/0-phase cells amounting to approximately 95% of gated cells after doxycycline treatment of CEM 6E2 cells, which was accompanied by a decrease in S-phase cells (Fig. 7, lower left panel and S5 Fig.). CEM 2C8 cells did not show any significant alteration in cell cycle phase distribution with or without doxycycline treatment. Treatment of both cell lines with AMF under doxycycline-stimulated conditions did not reveal any difference between the tested CEM subclones 6E2 and 2C8 (Fig. 7, lower right panel and S6 Fig.), suggesting that also in G1/0-phase arrested cells, AMF induces apoptosis to the same extent as in proliferating cells. To the authors’ knowledge, this is a novel finding, as generally G1/0 arrest by itself induces apoptosis, and this has also been shown to be the case in CEM cells [36]. However, we show that in G1/0 arrested cells—though considered as more resistant to anti-proliferative and pro-apoptotic substances—apoptosis is substantially induced and not significantly diminished compared to non-arrested cells. In general, classic chemotherapeutic agents, like alkylating agents, topoisomerase II inhibitors or taxans induce apoptosis in a cell cycle dependent manner, being S- and M-phase as the most susceptible cell cycle phases. However, recently also others have found that quiescent chronic lymphocytic leukemia cells and also lung cancer stem cells are sensitive to apoptosis induction by the small sirtuin inhibitor Tenovin-6 [37] and the bcl-2/bcl-X_L_ inhibitor ABT-737 [27], respectively.

Taken together, in the context of current chemotherapeutic agents substances that induce apoptosis in proliferating and G1/0-arrested cells in a caspase-(in)dependent way would be of great clinical benefit.

### AMF reduces tumor growth *in vivo*

**Fig 8:**
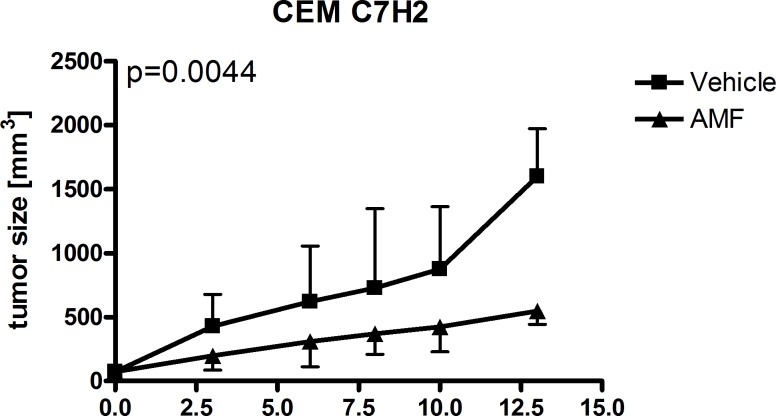
Jugular vein catheters were implanted in male nu/nu Balb/c mice. A matrigel CEM C7H2 1:1 mixture was injected into the flanks of male and mice were treated with AMF (5μMol/kg per day) for 13 days. Tumors size was measured regularly and tumors were harvested for immunohistochemistry on day 13. n = 4 per treatment group.

**Fig 9:**
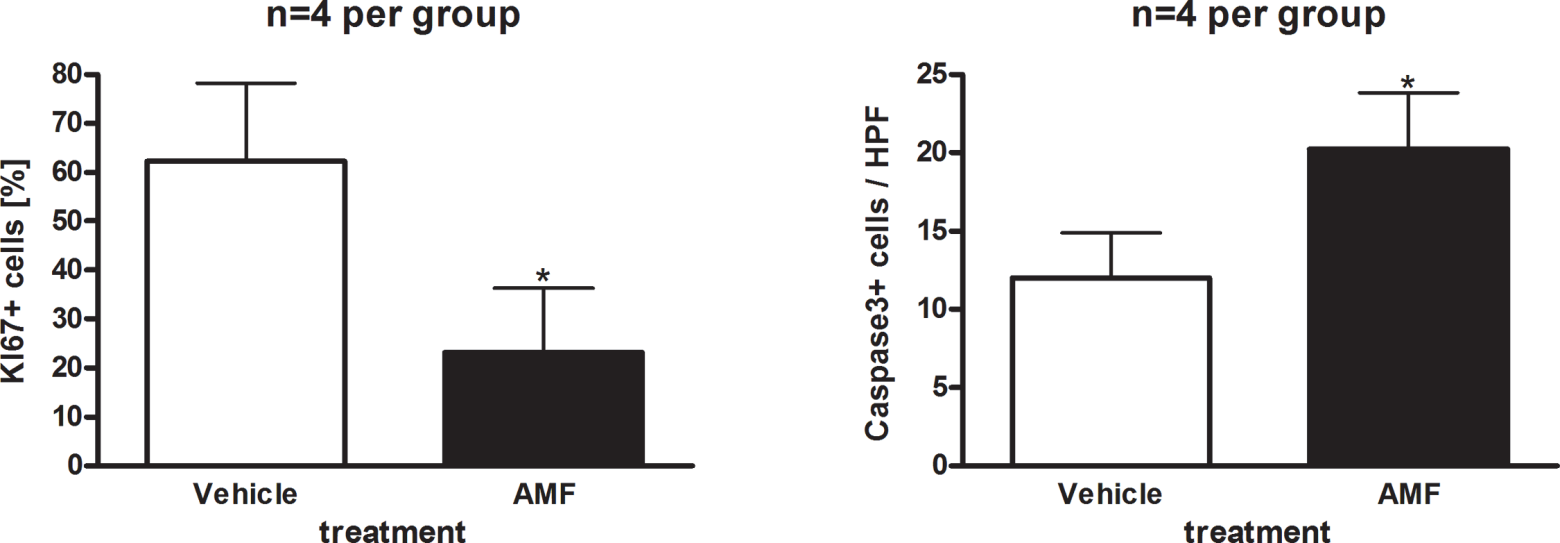
KI67 positive cells are reduced and caspase 3 is induced after AMF treatment. After 13 days of AMF treatment mice were killed and tumors were harvested for immunohistochemistry. Means + SD (n = 4 per treatment group) are shown.

**Fig 10:**
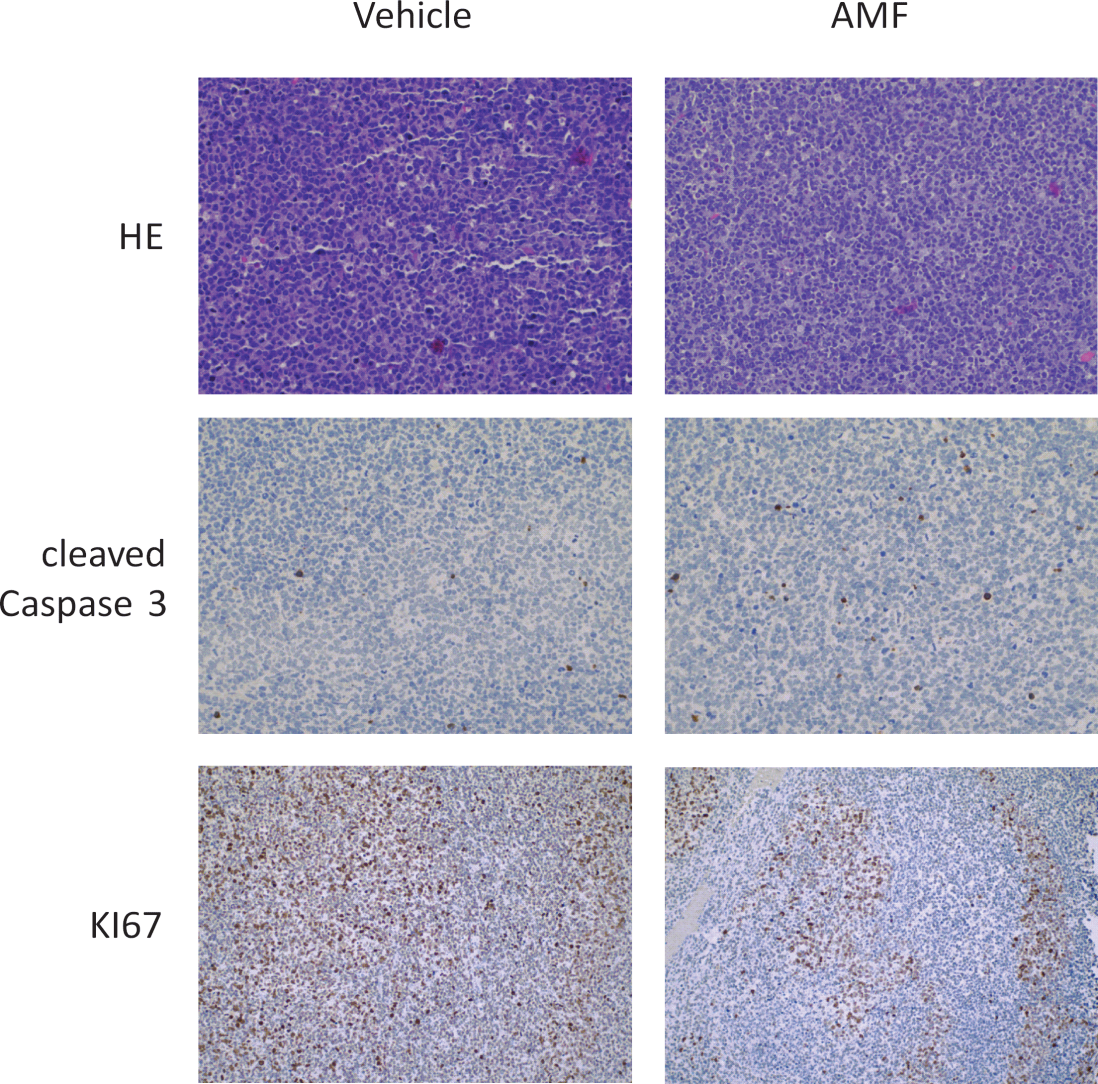
KI67 positive cells are reduced and caspase 3 is induced after AMF treatment. After 13 days of AMF treatment mice were killed and tumors were harvested for immunohistochemistry. Representative sections of control and treated tumors (hematoxyline-eosine staining, caspase 3 and KI67 staining) are shown.

The last step was to test AMF in vivo. For these experiments, we used Nu/Nu Balb/c mice which were injected subcutaneously with 1x10^6^ cells (1:1 mixed with matrigel, total volume 100 μl) after implantation of a jugular vein catheter. One week after injection, tumor was visible and we initiated treatment with AMF 5 mg/kg i.v.. As shown in Fig. 8, AMF caused significant inhibition of tumor growth. In hematoxylin eosin staining, the AMF-treated tumors appeared to have slightly smaller cells in general and had a lower cytoplasma:nucleus ratio compared to vehicle-treated tumors. Immunohistochemical analysis of KI67 and caspase 3 revealed a significantly lower KI67 and higher caspase 3 expressions in AMF-treated tumors (Figs. 9 and 10).

In conclusion, although we could not show a tumor size reduction over treatment time, we found that AMF significantly reduced tumor growth compared to vehicle-treated mice. One possible explanation for only a growth-inhibitory effect might be that we have used only 5 mg/kg bodyweight. Although this is twice as much as published to be well tolerated [38], we were cautious because of recent data where 10mM (equal app. to 10mg/liter) was detrimental for zebrafish in vivo [39]. Given the fact that earlier studies with a similar compound Akacid plus was tested successfully at a concentration of 200mg/kg (LD50 2000mg/kg) in female rats [40], one might hypothesize that higher concentrations would have yielded a more substantial anti-proliferative effect or even tumor size reduction.

In conclusion, we show that AMF has anti-proliferative and pro-apoptotic effects on the four leukemic cell lines (CCRF-CEM, K-562, U-937, HL-60) tested. In comparison with cell lines derived from normal lymphocytes malignant cells were more sensitive to AMF. Apoptosis induction was associated with activation of both caspase-dependent and caspase–independent pathways in proliferating and G1/0 arrested cells. In vivo, we found a substantial growth retardation of CEM cells in the AMF-treated group of mice, which was associated with increased expression of caspase-3 and decreased expression of the proliferation marker KI67. We believe that these results warrant further studies for elucidating AMF-induced apoptosis in detail in vitro and in vivo. In particular, as chemotherapy patients are susceptible to bacterial and fungal infections and AMF also has bactericidal and anti-fungal properties [40,41,42,43], additional research in this area can be expected to provide insights which might be clinically useful.

## Supporting Information

S1 Fig The pancaspase inhibitor Z-VAD-fmk significantly inhibits FasL induced apoptosis in CEM C7H2 cells. n = 3 (number of independent experiments carried out in triplicates).
(TIF)

S2 Fig In order to functionally test bcl-2 effects on apoptosis, CEM subclones with tetracycline-responsive bcl-2 expression (TET-off; 10E1+ doxycycline) and controls (10E1- without doxycycline) were treated with dexamethason. Representative dot-plots (FL1-H (Annexin-V-FITCS) against FL3-H (propidium-iodide)) are shown.
(TIF)

S3 Fig In order to functionally test CrmA effects on apoptosis, CEM subclones with constitutive expression of CrmA (2E8) and controls (D2.1) were treated with FasL and C2-ceramid. Representative dot-plots (FL1-H (Annexin-V-FITCS) against FL3-H (propidium-iodide)) are shown.
(TIF)

S4 Fig CEM subclones with tetracycline-responsive bcl-2 expression (TET-off; 10E1+) and constitutive expression of CrmA (2E8) and their respective controls (10E1-, D2.1) were treated with different concentrations of AMF (as indicated on the y-axis). Representative dot-plots (FL1-H against FL3-H) are shown.
(TIF)

S5 Fig CEM 6E2 and 2C8 cells were treated with doxycyline (+) or not (-). CEM 6E2 cells express the cell cycle inhibitor p16^INK4A^ upon doxycycline treatment and are arrested in G1/0-phase. Representative flowcytometric analyses are shown.
(TIF)

S6 Fig CEM 6E2 (G1/0-arrested) and 2C8 (proliferating) cells were treated with doxycyline and different concentrations of AMF (stated on the y-axis). In both cell lines apoptosis is induced to a similar extend. Representative dot-plots (FL1-H against FL3-H) are shown.
(TIF)
