# Supplementary figures and images for: Akacid Medical Formulation Induces Apoptosis in Myeloid and Lymphatic Leukemic Cell Lines In Vitro and In Vivo
Source: PLoS One. 2015 Feb 13;10(2):e0117806. doi: 10.1371/journal.pone.0117806 (PMC4334520; doi:10.1371/journal.pone.0117806)

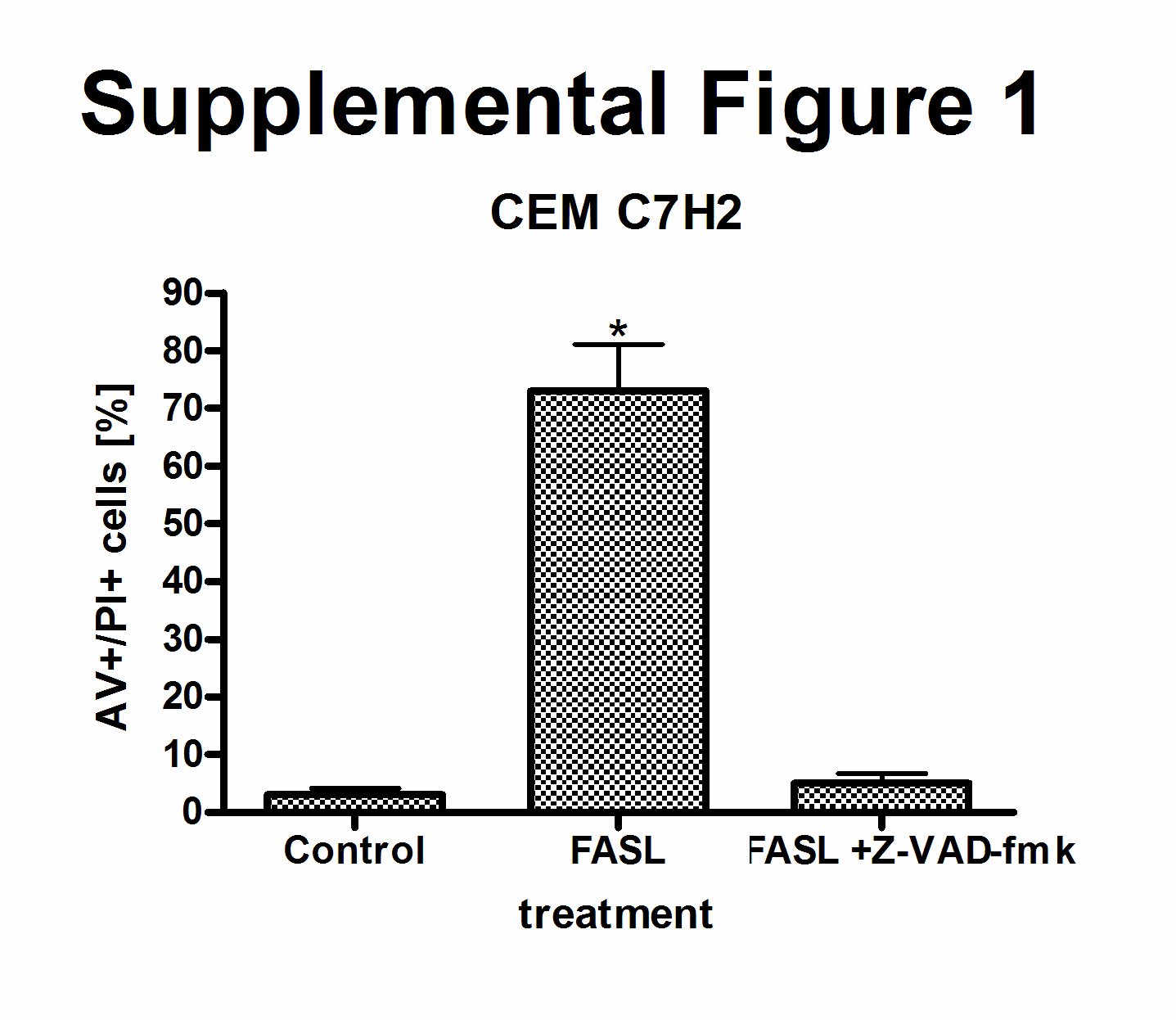

Supplement: S1 Fig — n = 3 (number of independent experiments carried out in triplicates). (TIF) [file pone.0117806.s001.tif]

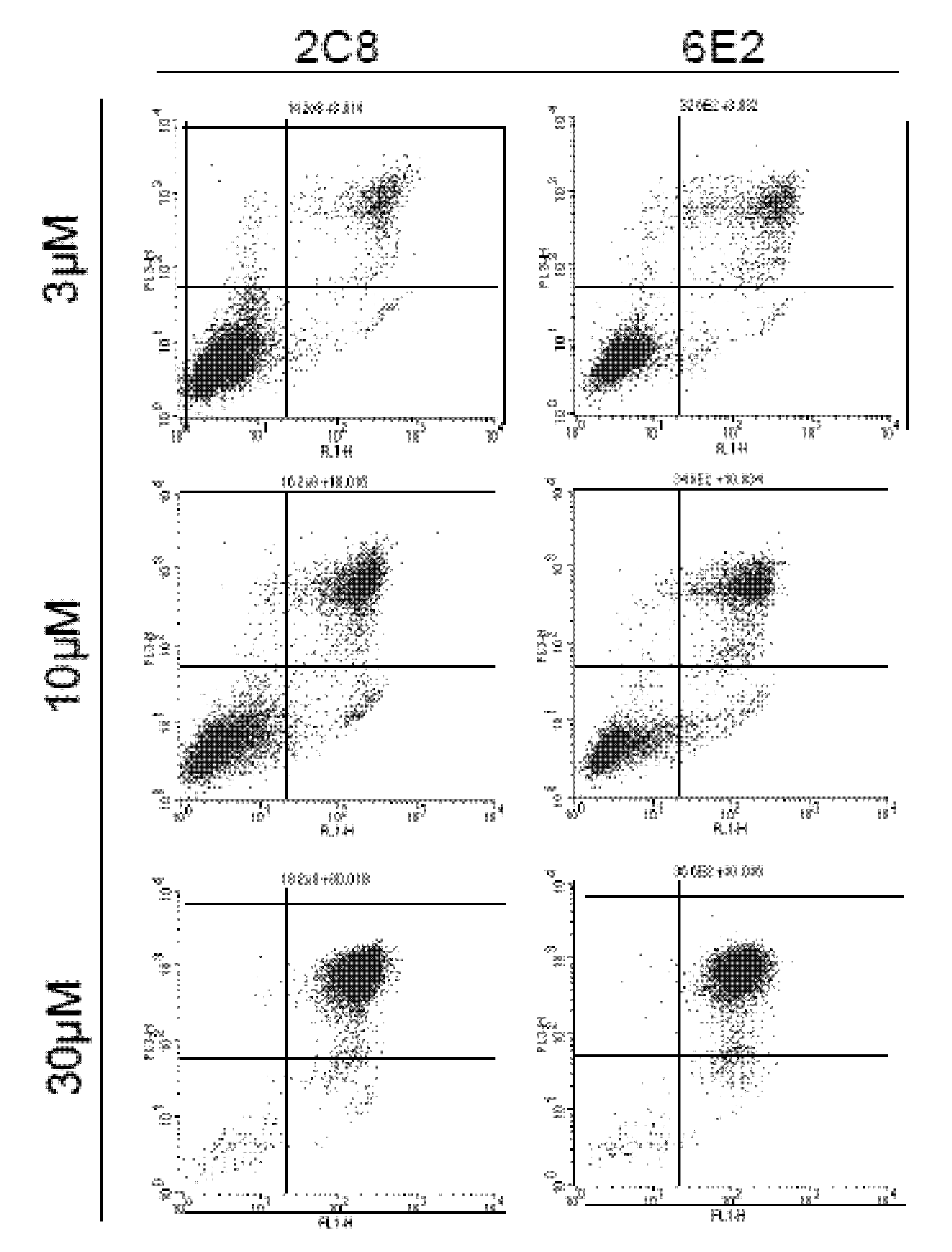

Supplement: S6 Fig — In both cell lines apoptosis is induced to a similar extend. Representative dot-plots (FL1-H against FL3-H) are shown. (TIF) [file pone.0117806.s006.tif]
